# Real-World Data on Ramucirumab Therapy including Patients Who Experienced Two or More Systemic Treatments: A Multicenter Study

**DOI:** 10.3390/cancers14122975

**Published:** 2022-06-16

**Authors:** Yutaka Yasui, Masayuki Kurosaki, Kaoru Tsuchiya, Yuka Hayakawa, Chitomi Hasebe, Masami Abe, Chikara Ogawa, Kouji Joko, Hironori Ochi, Toshifumi Tada, Shinichiro Nakamura, Koichiro Furuta, Hiroyuki Kimura, Keiji Tsuji, Yuji Kojima, Takehiro Akahane, Takashi Tamada, Yasushi Uchida, Masahiko Kondo, Akeri Mitsuda, Namiki Izumi

**Affiliations:** 1 Department of Gastroenterology and Hepatology, Musashino Red Cross Hospital, Tokyo 180-8610, Japan; yutakay@musashino.jrc.or.jp (Y.Y.); kurosaki@musashino.jrc.or.jp (M.K.); tsuchiya@musashino.jrc.or.jp (K.T.); y.hayakawa@musashino.jrc.or.jp (Y.H.); 2 Department of Gastroenterology, Japanese Red Cross Asahikawa Hospital, Asahikawa 070-8530, Japan; hasebe@asahikawa-rch.gr.jp (C.H.); masami@asahikawa-med.ac.jp (M.A.); 3 Department of Gastroenterology, Takamatsu Red Cross Hospital, Takamatsu 760-0017, Japan; chikara.ogawa.19721202@gmail.com; 4 Center for Liver-Biliary-Pancreatic Disease, Matsuyama Red Cross Hospital, Matsuyama 790-8524, Japan; koujijoko@matsuyama.jrc.or.jp (K.J.); hironori19810211@matsuyama.jrc.or.jp (H.O.); 5 Department of Gastroenterology, Japanese Red Cross Society Himeji Hospital, Himeji 670-8540, Japan; tadat0627@gmail.com (T.T.); s-nakamura@himeji.jrc.or.jp (S.N.); 6 Department of Internal Medicine, Masuda Red Cross Hospital, Masuda 698-8501, Japan; furuta-k@masuda.jrc.or.jp; 7 Department of Gastroenterology, Japanese Red Cross Kyoto Daiichi Hospital, Kyoto 605-0981, Japan; hiroyuki-kimura@kyoto1-jrc.org; 8 Department of Gastroenterology, Hiroshima Red Cross Hospital and Atomic-bomb Survivors Hospital, Hiroshima 730-8619, Japan; k-tsuji@hiroshima-med.jrc.or.jp; 9 Department of Gastroenterology, Japanese Red Cross Ise Hospital, Ise 516-8512, Japan; tdim_yrch@ise.jrc.or.jp; 10 Department of Gastroenterology, Ishinomaki Red Cross Hospital, Ishinomaki 986-8522, Japan; akahane-ttyh@mva.biglobe.ne.jp; 11 Department of Gastroenterology, Takatsuki Red Cross Hospital, Takatsuki 569-1096, Japan; ttam@bc4.so-net.ne.jp; 12 Department of Gastroenterology, Matsue Red Cross Hospital, Matsue 690-8506, Japan; laboratory@matsue.jrc.or.jp; 13 Department of Gastroenterology, Otsu Red Cross Hospital, Otsu 520-8511, Japan; masachan44@gmail.com; 14 Department of Internal Medicine, Japanese Red Cross Tottori Hospital, Tottori 680-8517, Japan; akeri@tottori-med.jrc.or.jp

**Keywords:** ramucirumab, molecularly targeted agents, hepatocellular carcinoma

## Abstract

**Simple Summary:**

Ramucirumab has been shown to be effective as a second-line agent after sorafenib in hepatocellular carcinoma (HCC) patients whose α-fetoprotein was ≥400 ng/mL. We performed a retrospective cohort study to investigate ramucirumab efficacy in a real-world setting. Progression-free survival (PFS) was consistent through treatment lines, modified albumin–bilirubin (mALBI) grade, Barcelona Clinic for Liver Cancer (BCLC) stage, and α-fetoprotein (AFP) level. By contrast, ascites was more frequently seen in mALBI 2b/3 patients and, therefore, should be carefully monitored.

**Abstract:**

Background: The present study aimed to clarify the efficacy and safety of ramucirumab in a real-world setting, including patients who experienced two or more systemic treatments or whose hepatic reserve was deteriorated. Methods: In total, 79 patients with hepatocellular carcinoma (HCC) from 14 institutes throughout Japan were retrospectively analyzed. The response was evaluated using the Response Evaluation Criteria in Solid Tumors (RECIST) version 1.1, and AEs were recorded according to the Common Terminology Criteria for AEs (CTCAE) version 5.0. Results: Median overall survival (OS) in the total cohort was 7.5 months (m). Median OS was 8.8 m in patients who were administered ramucirumab as a second-line treatment, while it was 7.3 m in third- or later-line treatment. Progression-free survival rates in the second- and third- or later-line therapies were 3.2 m and 3.2 m, respectively. The disease control rate (DCR) in the study was 43%. There were no statistically significant differences in DCR between the treatment courses. Regarding adverse events (AEs), the development of ascites was observed significantly more frequently in modified albumin–bilirubin (mALBI) 2b/3 patients than in mALBI 1/2a patients (54.5% vs. 25.0%, *p* = 0.03). Conclusions: Ramucirumab is useful as a second-line therapy and feasible as a third- or later-line treatment for HCC.

## 1. Introduction

The treatment of hepatocellular carcinoma (HCC) has seen rapid development [1]. In the past few years, first-line therapies [2], as well as several second-line treatments, have been newly introduced [3]. One of the second-line agents is ramucirumab, which is a human monoclonal antivascular endothelial growth factor receptor-2 antibody. In the REACH trial, ramucirumab initially showed its efficacy in a subgroup of HCC patients who have high α-fetoprotein (AFP) levels [4]. Later, the REACH-2 trial [5] was conducted as the second-line setting after sorafenib, enrolling only patients with AFP levels of ≥400 ng/mL. Consequently, the REACH-2 study showed that ramucirumab significantly prolonged survival when compared with placebo. Following these results, ramucirumab was approved for second-line treatment for HCC patients with AFP levels of ≥400 ng/mL.

Presently, a combination of atezolizumab plus bevacizumab is the first choice of first-line treatment [6]. For patients for whom this treatment can not apply, sorafenib [7] and lenvatinib [8] are chosen as the first-line molecularly targeted agents (MTA). As second-line treatments, regorafenib [9] and cabozantinib [10] are available besides ramucirumab, whereas both sorafenib and lenvatinib, which were not administered as the first line, are also being used. Although the evidence was initially presented as the second-line treatment after sorafenib, these drugs are chosen as the second- or further-line treatment after lenvatinib. In addition, ramucirumab was reported not to impact liver function negatively [11]. Given this situation, there are still many questions to be answered with real-world clinical data for patients who experienced two or more systemic therapies, whose liver function has deteriorated, or who have comorbidities. The real-world data of systemic therapy have been highlighted and revealed many significant findings that could not be clarified in clinical trials [12,13,14,15,16]. The present study aimed to determine the optimal sequential treatment for HCC patients and the treatment strategy of ramucirumab treatment.

## 2. Materials and Methods

### 2.1. Patients

Patients treated with ramucirumab for intermediate or advanced stage HCC from 14 hospitals throughout Japan were identified from the clinical database. All patients had received at least one MTA treatment before receiving ramucirumab. Patients who were administered ramucirumab for at least two cycles were analyzed. We retrospectively collected baseline characteristics and clinical data during ramucirumab treatment, including information on any prior treatment course, the Eastern Cooperative Oncology Group performance status (ECOG-PS), Barcelona Clinic for Liver Cancer (BCLC) stage, tumor burden, and hepatic reserve evaluated by Child–Pugh grade, as well as albumin–bilirubin (ALBI) grade. Baseline AFP was ≥400 ng/mL in all the patients. Written informed consent was received from each patient for the ramucirumab treatment; however, the requirement for informed consent for the study was waived because of its retrospective nature. This study was conducted in accordance with the Declaration of Helsinki, and the institutional ethical committee of Musashino Red Cross Hospital approved the study protocol (approval number: 2039).

### 2.2. Treatment Procedures

Ramucirumab (8 mg/kg) was administered intravenously every two weeks. Dose reduction and treatment discontinuation of ramucirumab was decided per the information prescribed by the manufacturer. An initial dose modification was allowed when the attending physicians deemed it necessary for any appropriate reason, such as proteinuria, due to prior therapy and/or patient comorbidity.

### 2.3. Outcomes

Overall survival (OS) was defined as the time from the first administration of ramucirumab to the date when the patient died due to any cause or the date of the last visit. The initial imaging assessment was performed at 8 ± 2 weeks after the initiation of ramucirumab administration, followed by every 2–3 months. The response was evaluated using the Response Evaluation Criteria in Solid Tumors (RECIST) version 1.1 [17]. Progression-free survival (PFS) was defined as the time from the first drug administration to the date when the progression or any cause of death was recorded or the date of the last radiological evaluation. AEs were recorded according to the Common Terminology Criteria for AEs (CTCAE) version 5.0. The results were analyzed in total enrolled patients, and subgroups were stratified by the treatment course of ramucirumab or liver function reserve.

### 2.4. Statistical Analysis

Continuous variables are provided as medians with interquartile ranges and compared using the Mann–Whitney U test or Kruskal–Wallis test. Categorical variables are given as numbers and percentages and compared using Fisher’s exact test. Survival data were analyzed using the Kaplanؘ–Meier method and compared using a log-rank test. A probability (*p*) value of <0.05 was considered statistically significant. All statistical analyses were conducted using the graphical interface of R (EZR) [18].

## 3. Results

### 3.1. Patients

Table 1 shows the baseline characteristics of the patients enrolled in the present study. A total of 79 patients with a median age of 71 from 14 institutes across Japan were enrolled in this study. Among these, 17 (21.5%) were females, and hepatitis C was the most frequent cause of background liver disease. Regarding the treatment course, ramucirumab was administered as the second-, third-, fourth-, and fifth-line treatments in 40 (50.6%), 13 (16.4%), 24 (30.3%), and 2 (2.5%) patients, respectively. Among 40 patients who were administered ramucirumab as second-line therapy, 34 had received lenvatinib as the first-line treatment, while 4 had received sorafenib. Forty-four patients (55.7%) were classified as ECOG-PS 1, whereas only two patients were ECOG-PS 2. Hepatic reserve at Child–Pugh grade A was seen in 44 (55.7%) patients, and the Child–Pugh score was ≤7 in 63 (79.7%) patients. Modified albumin–bilirubin (mALBI) grade 1 or 2a was seen in 24 (30.4%). Overall, 40 (50.6%) patients were classified as BCLC stage C, 39 (49.4%) of whom had extrahepatic spread, and 27 (34.2%) had a major vascular invasion. The median observational time was 6.3 months.

Regarding previous treatment before ramucirumab, 53 (67%) patients had received lenvatinib as the first-line therapy, whereas sorafenib had been administered as first-line therapy for 22 (28%) patients. Lenvatinib was administered at any line of treatment in 73 patients before ramucirumab. Atezolizumab plus bevacizumab combination had administered in six patients before ramucirumab. In Table 2, the data on treatment history before ramucirumab therapy are summarized.

### 3.2. Overall Survival (OS)

**Figure 1:**
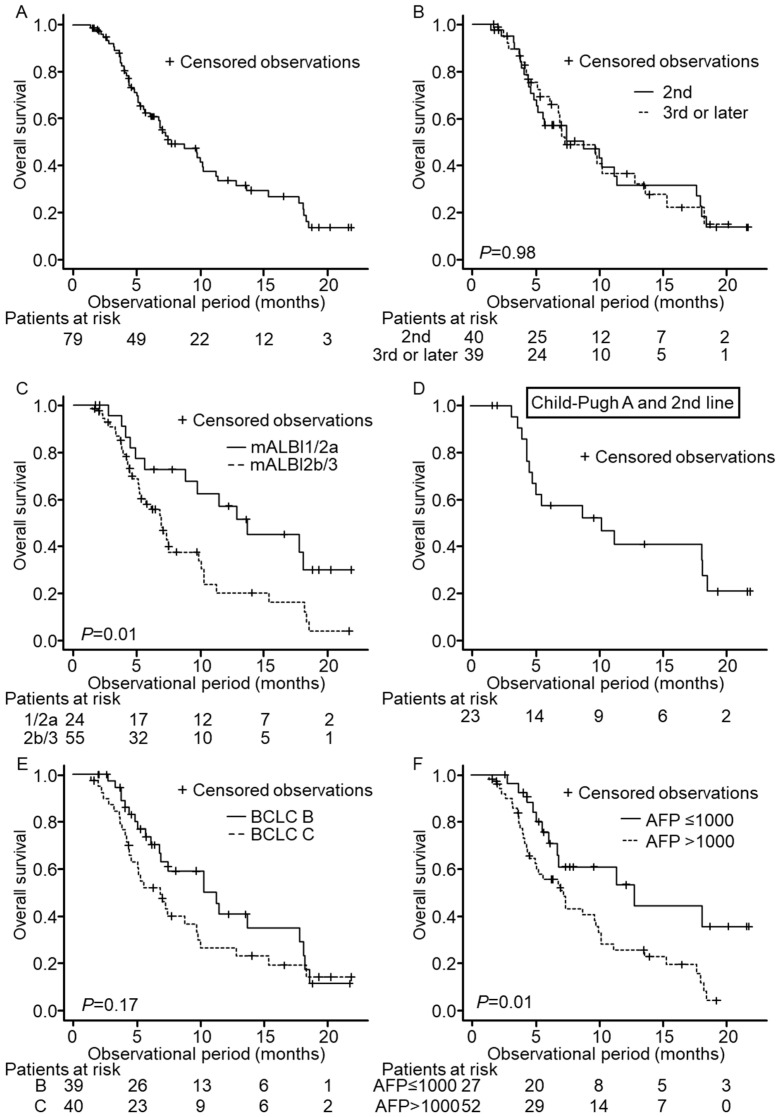
Overall survival (OS) of patients enrolled in this study. Censored observations are marked as + in each Kaplan–Meier curve. Each panel shows OS in the entire cohort (**A**), OS according to the treatment course of ramucirumab (**B**), mALBI grade (**C**), BCLC stage (**E**), and AFP level (**F**), respectively. Panel (**D**) shows OS in patients of 2nd-line treatment with Child–Pugh A.

In total, 51 patients out of 79 died during the follow-up period. The median overall survival (OS) was 7.5 months (Figure 1A). In terms of the treatment course, the OS of patients with second-line treatment patients was not significantly different from patients with third- or later-line treatment (median OS 8.8 months vs. 7.3 months, *p* = 0.98; Figure 1B). With regard to hepatic reserve, OS of Child–Pugh A patients (*n* = 44) tended to show longer survival than that of Child–Pugh B/C patients (*n* = 35) (median OS 10.3 months vs. 6.7 months, *p* = 0.06). OS of mALBI grade 1/2a patients was significantly longer than that of mALBI 2b/3 patients (median OS 13.6 months vs. 6.9 months, *p* = 0.01, Figure 1C). To compare with the REACH-2 trial, we analyzed 23 patients who were Child–Pugh A at baseline and administered ramucirumab as second-line treatment. The median OS of this subgroup was 10.3 months (Figure 1D), which was comparable to the median OS of the REACH-2 trial, with 8.5 months. According to BCLC stage (Figure 1E), OS was not significantly different between BCLC stage B and C (11.2 months vs. 6.9 months, *p* = 0.17), whereas OS whose baseline AFP > 1000 ng/mL showed significantly shorter survival than those with baseline AFP ≤ 1000 ng/mL (Figure 1F, 12.8 months vs. 7.3 months, *p* = 0.01).

### 3.3. Progression-Free Survival (PFS) and Radiological Response

**Figure 2:**
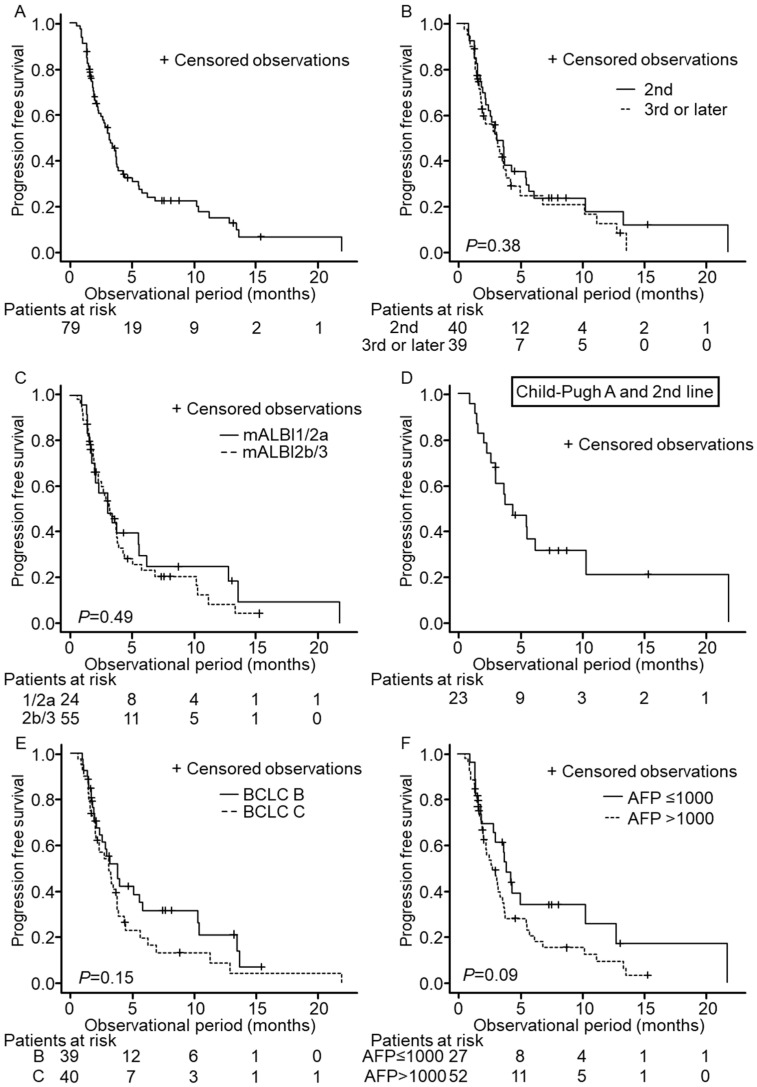
Progression-free survival (PFS) of patients enrolled in this study. Censored observations are marked as + in each Kaplan–Meier curve. Panel (**A**) shows PFS in the entire cohort. PFS according to the treatment course of ramucirumab (**B**), mALBI grade (**C**), BCLC stage (**E**), and AFP level (**F**) are presented. Panel (**D**) shows PFS in patients of 2nd-line treatment with Child–Pugh A.

PFS was 3.2 months in all patients (Figure 2A), and no significant difference was seen between PFS in second- and third- or later-line treatment (median PFS 3.2 m vs. 3.2 m, *p* = 0.38, Figure 2B). Furthermore, no significant difference was observed between mALBI grade 1/2a and 2b/3, BCLC stage B and C, or baseline AFPs ≤ 1000 ng/mL and >1000 ng/mL (Figure 2C,E,F). Median PFS of Child–Pugh A patients who were administered ramucirumab as second-line was 4.4 months (Figure 2D). Again, this result was comparable to the median PFS of the REACH-2 trial, with 2.8 months. Among the enrolled patients, 65 patients underwent at least 1 radiological assessment, and we observed radiological progression in 41 patients during the follow-up period. Best response as identified by RECIST ver. 1.1 was PR in 1 patient and SD in 27 patients, and the disease control rate (DCR) was 43.0%. DCR was not significantly different between second-line treatment and third- or later-line treatment.

### 3.4. Adverse Events (AEs)

An AE at any grade was observed in 73 (92.4%) patients. A grade 3 AE was recorded in 20 (25.3%) patients, and 16 (20.2%) patients discontinued ramucirumab due to a treatment-related AE. Table 3 lists all the AE observed in the present study. The most frequent AE was hypertension, followed by fatigue and appetite loss, most of which were grade 1 or 2. The development or worsening of ascites was observed in 36 patients, and 8 of those did not undergo any additional treatment, whereas 28 were managed by diuretics. The most frequent grade 3 AE was peripheral edema in nine patients, followed by ascites in four patients. Neither the frequency of any grade AE nor grade 3 AE was significantly different between the treatment courses. There was no statistically significant difference in the frequency of any grade AE or any grade 3 AE between Child–Pugh A and Child–Pugh B/C patients. However, peripheral edema, ascites, and diarrhea were significantly more frequent in Child–Pugh B/C patients than in Child–Pugh A patients (peripheral edema; 57.1% vs. 31.8%, *p* = 0.04, ascites; 68.6% vs. 27.3%, *p* < 0.001, diarrhea; 25.7% vs. 4.5%, *p* = 0.009). Ascites was significantly more frequent in mALBI 2b/3 patients than in mALBI 1/2a patients (54.5% vs. 25.0%, *p* = 0.03).

### 3.5. Change in AFP during Ramucirumab Treatment and Subsequent Treatment after Ramucirumab

During ramucirumab therapy, we observed AFP dynamics in 72 patients. AFP decrease of over 20% at week four was seen in nine patients, while AFP increased by less than 20% in 19 patients. Patients without over 20% increase in AFP at week 4 showed significantly longer PFS than those with over 20% increase (median PFS 3.8 vs. 2.3 months, *p* = 0.02). Furthermore, 63 patients were followed up after ramucirumab. Thirty-five (55.6%) received subsequent treatment, including 19 patients who received systemic treatment and seven patients who received transarterial chemoembolization (TACE). Patients who received subsequent treatment after radiological progression had longer survival than those who did not (10.2 months vs. 6.8 months, *p* = 0.005). Patients with baseline mALBI grade 1/2a had a significantly higher probability of subsequent treatment than patients with mALBI 2b/3 (83.3% vs. 44.4%, *p* = 0.006).

## 4. Discussion

The present study provides real-life data on ramucirumab-treated patients, including those who had received two or more MTA treatments before. Our data also demonstrated that ramucirumab treatment was effective and safe for patients who had received lenvatinib or other MTAs. PFS was consistent through treatment lines, mALBI grade, BCLC stage, and AFP level. In patients with a deteriorated liver function such as Child–Pugh B or mALBI 2b/3, several AEs were more frequent than those whose liver function was preserved. Therefore, AE monitoring and management are essential in Child–Pugh B or mALBI 2b/3 patients. Our data suggest that ramucirumab treatment can expect consistent PFS in a wide range of patients. After the approval of atezolizumab and bevacizumab combination therapy, MTAs sequential therapy has become critical for HCC patients [19,20,21,22,23]. There are still clinical questions such as optimal sequential usage of available systemic treatments. 

REACH and REACH-2 trials were conducted in the second-line setting after sorafenib for Child–Pugh A patients; however, there was little evidence about the efficacy and safety of ramucirumab for patients after lenvatinib or multiple lines of treatment. In our study, 85% of second-line patients, and 90% of all patients had a treatment history of lenvatinib. Regarding the efficacy, the OS of Child–Pugh A patients treated with second-line ramucirumab therapy was comparable to the result of the REACH-2 trial. Kuzuya et al. first reported on the outcome of ramucirumab treatment after lenvatinib and provided similar observations in 12 patients [24]. Hiraoka et al. reported the efficacy of ramucirumab treatment as second- to fourth-line after lenvatinib in 28 patients [25], which provided evidence of ramucirumab in third- or later-line treatment. Our data confirmed these reports in a larger cohort. Regarding safety, the frequency of any grade AE and grade 3 AE in our cohort was not significantly different from that of the REACH or the REACH-2 trial, suggesting that the safety profile would not vary in patients who had received lenvatinib. Kasuya et al. also reported no serious AE in seven patients who received ramucirumab as the second or third line after lenvatinib [26]. These results suggest that ramucirumab can be used safely even after lenvatinib.

The subanalyses of the clinical trial clarified the survival benefits of ramucirumab in the elderly [27] and the Japanese subgroup [28,29]. In contrast, we focused on the efficacy of ramucirumab as the third- or later-line therapy. Our data revealed no statistical differences between the second- and third- or later-line therapy in the OS or PFS. OS in our cohort was shorter than that of the REACH-2 trial. This result could be attributed to the baseline hepatic reserve since the REACH-2 trial only included Child–Pugh A patients, while the cohort in the present study comprised 38.2% Child–Pugh B patients. The survival benefit for Child–Pugh B patients was also marginal in an exploratory analysis of the REACH trial [30]. It should be noted that the evidence of clinical benefit of ramucirumab for Child–Pugh B patients is still not enough. Baseline hepatic reserve is a critical factor for the treatment strategy of unresectable HCC patients [31,32] and a predictive factor for patients whether they can be a candidate for next-line treatment [33,34]. In the present study, subsequent treatment was significantly more frequent in mALBI 1/2a patients. Therefore, treatment strategy during preserved liver function is essential. Meanwhile, the PFS was consistent regardless of treatment history, BCLC stage, and mALBI grade. In terms of AE, the development or worsening of ascites and peripheral edema was significantly more common in Child–Pugh B/C patients. In a recent study, Kudo et al. reported that ascites were not unfavorable AE in ramucirumab treatment [35]. For Child–Pugh B/C patients, careful observation and appropriate management for ascites should be considered. 

Our study has several limitations. The retrospective study design and the short duration of the follow-up period are major limitations. The median observational time of this study was relatively short at 6.3 months; therefore, analyses after prolonged follow-up should be considered. Additionally, because of the study design, which relied on data collection from many institutions, detailed data are lacking. A larger cohort with a longer follow-up period and detailed data are needed to confirm and further elucidate our results.

## 5. Conclusions

In conclusion, besides the second-line therapy, ramucirumab treatment was feasible as a third- or later-line treatment for hepatocellular carcinoma. The PFS using ramucirumab in the real-world setting, including patients administered ramucirumab after lenvatinib, was comparable with the PFS observed in the REACH-2 trial.

## Figures and Tables

**Table 1:** Baseline characteristics.

Factor	Group	*n* = 79
Age, median [IQR]		71 [66, 78]
Gender, *n* (%)	Female/Male	17 (21.5)/62 (78.5)
Etiology, *n* (%)	HCV/HBV/ Alcohol/Others	36 (45.6)/13 (16.5)/ 12 (15.2)/18 (22.8)
ECOG-PS, *n* (%)	0/1/2	33 (41.8)/44 (55.7)/2 (2.5)
Treatment course, *n* (%)	2nd line/3rd or later line	40 (50.6)/39 (49.4)
Child–Pugh grade, *n* (%)	A5/A6/B or C	14 (17.7)/30 (38.0)/35 (44.3)
mALBI grade, *n* (%)	1 or 2a/2b or 3	24 (30.4)/55 (69.6)
ALBI score, median [IQR]		−2.00 [−2.36, −1.67]
AST (U/L), median [IQR]		45 [33, 64]
ALT (U/L), median [IQR]		27 [20, 40]
Albumin (g/dL), median [IQR]		3.2 [2.8, 3.7]
Bilirubin (mg/dL), median [IQR]		0.8 [0.6, 1.1]
PT (%), median [IQR]		93 [79, 103]
BCLC stage, *n* (%)	B/C	39 (49.4)/40 (50.6)
Extrahepatic spread, *n* (%)	present/absent	39 (49.4)/40 (50.6)
Major vascular invasion, *n* (%)	present/absent	27 (34.2)/52 (65.8)
AFP (ng/mL), median [IQR]		1818 [743, 5679]
DCP (mAU/mL), median [IQR]		3166 [248, 14,631]
Duration of systemic therapy (days), median [IQR]		350 [126, 735]

Abbreviations: ECOG-PS; Eastern Cooperative Oncology Group performance status, BMI; body mass index, mALBI; modified albumin–bilirubin, AST; aspartate aminotransferase, ALT; alanine aminotransferase, eGFR; estimated glomerular filtration rate, PT; prothrombin time, WBC; white blood cell, BCLC; Barcelona Clinic for Liver Cancer, AFP; α-fetoprotein, DCP; des-gamma-carboxy prothrombin.

**Table 2:** Treatment history according to the number of regimens before ramucirumab.

1 *n* = 40	2 *n* = 13	3 *n* = 24	4 *n* = 2
Len 34	Len-Sor 5	Sor-Reg-Len 10	Len-Sor-Reg-A+B 1
Sor 4	Sor-Len 4	Len-Sor-Reg 7	Len-Sor-A+B-Len 1
CT 2	Sor-Reg 2	Len-A+B-Sor 2	
	Len-Reg 1	Len-Sor-Len 1	
	Len-A+B 1	Sor-Len-Reg 1	
		Sor-Len-Sor 1	
		CT-Len-A+B 1	
		CT-CT-Len 1	

Abbreviations: Len; lenvatinib, Sor; sorafenib, CT; clinical trial, Reg; regorafenib, A+B; atezolizumab plus bevacizumab combination therapy.

**Table 3:** Adverse events during ramucirumab treatment.

	Any Grade AE	Grade 3 AE	2nd (*n* = 40)	3rd or later (*n* = 39)	*p* Value	mALBI 1/2a (*n* = 24)	mALBI 2b/3 (*n* = 55)	*p* Value	Child–Pugh A (*n* = 44)	Child–Pugh B/C (*n* = 35)	*p* Value
Hypertension	41 (51.9)	1 (1.3)	22 (55.0)	19 (48.7)	0.66	16 (66.7)	25 (45.5)	0.09	28 (63.6)	13 (37.1)	0.02
Fatigue	38 (48.1)	2 (2.5)	22 (55.0)	16 (41.0)	0.26	12 (50.0)	26 (47.3)	>0.99	20 (45.5)	18 (51.4)	0.65
Appetite loss	38 (48.1)	0 (0.0)	20 (50.0)	18 (46.2)	0.82	12 (50.0)	26 (47.3)	>0.99	19 (43.2)	19 (54.3)	0.37
Ascites	36 (45.6)	4 (5.1)	18 (45.0)	18 (46.2)	>0.99	6 (25.0)	30 (54.5)	0.03	12 (27.3)	24 (68.6)	<0.001
Proteinuria	35 (44.3)	2 (2.5)	15 (37.5)	20 (51.3)	0.26	11 (45.8)	24 (43.6)	>0.99	21 (47.7)	14 (40.0)	0.51
Peripheral edema	34 (43.1)	9 (11.4)	13 (32.5)	21 (53.8)	0.07	9 (37.5)	25 (45.5)	0.62	14 (31.8)	20 (57.1)	0.04
Elevation of AST or ALT	13 (16.5)	0 (0.0)	6 (15.0)	7 (17.9)	0.77	5 (20.8)	8 (14.5)	0.52	8 (18.2)	5 (14.3)	0.76
Nausea	11 (13.9)	0 (0.0)	4 (10.0)	7 (17.9)	0.35	4 (16.7)	7 (12.7)	0.73	7 (15.9)	4 (11.4)	0.75
Diarrhea	11 (14)	1 (1.3)	5 (12.5)	6 (15.4)	0.76	1 (4.2)	10 (18.2)	0.16	2 (4.5)	9 (25.7)	0.009
Liver failure	10 (12.7)	1 (1.3)	6 (15.0)	4 (10.3)	0.74	2 (8.3)	8 (14.5)	0.72	4 (9.1)	6 (17.1)	0.33
Bleeding	6 (7.8)	3 (3.9)	2 (5.0)	4 (10.3)	0.43	2 (8.3)	4 (7.3)	>0.99	4 (9.1)	2 (5.7)	0.69
Elevation of total bilirubin	6 (7.6)	2 (2.5)	3 (7.5)	3 (7.7)	>0.99	1 (4.2)	5 (9.1)	0.66	3 (6.8)	3 (8.6)	>0.99
Fever	5 (6.3)	0 (0.0)	4 (10.0)	1 (2.6)	0.36	1 (4.2)	4 (7.3)	>0.99	1 (2.3)	4 (11.4)	0.17
Abdominal pain	4 (5.1)	1 (1.3)	2 (5.0)	2 (5.1)	>0.99	2 (8.3)	2 (3.6)	0.58	3 (6.8)	1 (2.9)	0.63
Heart failure	3 (3.8)	1 (1.3)	2 (5.0)	1 (2.6)	>0.99	1 (4.2)	2 (3.6)	>0.99	3 (6.8)	0 (0.0)	0.25
Thrombosis	2 (2.6)	0 (0.0)	0 (0.0)	2 (5.1)	0.24	1 (4.2)	1 (1.8)	0.52	1 (2.3)	1 (2.9)	>0.99
Infusion reaction	0 (0.0)	0 (0.0)	0 (0.0)	0 (0.0)	>0.99	0 (0.0)	0 (0.0)	>0.99	0 (0.0)	0 (0.0)	>0.99

Abbreviations: AST; aspartate aminotransferase, ALT; alanine aminotransferase, AE; adverse events.

## Data Availability

The datasets supporting the study conclusions are included within this manuscript.

## Abbreviations

Hepatocellular carcinoma	HCC
α-Fetoprotein	AFP
molecularly targeted agents	MTA
Adverse events	AEs
Eastern Cooperative Oncology Group performance status	ECOG-PS
Barcelona Clinic for Liver Cancer	BCLC
Modified albumin–bilirubin grade	mALBI grade
Overall survival	OS
Progression-free survival	PFS
Response Evaluation Criteria in Solid Tumors	RECIST
The Common Terminology Criteria for AEs	CTCAE
Disease control rate	DCR
